# Effect of Strain Rates and Heat Exposure on Polyamide (PA12) Processed via Selective Laser Sintering

**DOI:** 10.3390/ma16134654

**Published:** 2023-06-28

**Authors:** Chiara Morano, Marco Alfano, Leonardo Pagnotta

**Affiliations:** 1 Department of Mechanical, Energy and Management Engineering, University of Calabria, 87036 Rende, Italy; chiara.morano@unical.it; 2 Department of Mechanical and Mechatronics Engineering, University of Waterloo, 200 University Avenue West, Waterloo, ON N2L 3G1, Canada; marco.alfano@uwaterloo.ca

**Keywords:** polyamides, 3D printing, mechanical properties, thermal properties, nylon, strength

## Abstract

The use of polymers in the transportation industry represents a great opportunity to meet the growing demand for lightweight structures and to reduce polluting emissions. In this context, additive manufacturing represents a very effective fabrication route for mechanical components with sophisticated geometry that cannot be pursued by conventional methods. However, understanding the mechanical properties of 3D-printed polymers plays a crucial role in the performance and durability of polymer-based products. Polyamide is a commonly used material in 3D printing because of its excellent mechanical properties. However, the layer-by-layer deposition process and ensuing auxiliary steps (e.g., post-processing heating) may affect the microstructure and mechanical properties of 3D-printed nylon with respect to the bulk counterpart. In this work, we explore the effect of displacement rate and heat exposure on the mechanical properties of 3D-printed polyamide (PA12) specimens obtained by selective laser sintering (SLS). Moreover, the thermal characteristics of the powders and sintered material were evaluated using differential scanning calorimetry (DSC). Our results highlight the expected rate dependency of mechanical properties and show that a post-processing heat treatment partly affects mechanical behavior.

## 1. Introduction

Over the past decade, the growing demand to reduce CO_2_ emissions has led to the need for environmentally friendly processes and eco-friendly materials. Among them, thermoplastic polymers represent a great option since they could be reused or recycled. Several polymers fall into the category of thermoplastics, either amorphous or semi-crystalline, with a wide range of mechanical properties [1]. These materials can be recycled multiple times as they are not subject to thermal degradation [2] and can be reused for the manufacture of new components [2,3,4,5]. Moreover, thermoplastic polymers can be processed using 3D printing [6], i.e., one of the most attractive manufacturing technologies since it allows the fabrication of complex parts in a fairly simple way. According to ISO/ASTM 52900 [7], different processes fall under the additive manufacturing (AM) denomination. Among them, powder bed fusion (PBF) is one of the most versatile and earliest techniques that has been successfully employed for the fabrication of parts with different types of materials. This technology is defined as a “process in which thermal energy selectively fuses regions of a powder bed” [7]. According to the heat source used, it is possible to classify techniques into the following classes: (i) laser fusion (L), (ii) electron beam fusion (EB), and (iii) thermal fusion generally by infrared light (IrL). Moreover, the laser fusion processes can also be subdivided into selective laser sintering (SLS), and selective laser melting (SLM).

The selective laser sintering (SLS) process is widespread because of its many benefits, such as obtaining parts with complex shapes, good dimensional accuracy, and surface quality. Different studies have demonstrated the possibility of realizing complex structures using SLS technology without the need for additional machining processes [8,9,10]. As such, it is possible to reduce the time to market and, consequently, fabrication costs.

Polymers are widespread materials in additive manufacturing [11,12]; despite that, only some types are suitable for use in the selective laser sintering (SLS) process. Among these, even today, more than 90% of industrial consumption for SLS is limited to polyamide 12 (PA12) or its blends, e.g., dry blends of glass-filled or carbon-fiber-filled polyamides. Therefore, it is not surprising that most recent studies have focused on the analysis of PA12 [13,14,15,16,17]. El Magri et al. [13] evaluated the influence of laser power and hatching distance on mechanical and thermal properties. The authors found the material properties were mostly affected by laser power rather than by hatching distance. Martynková et al. [14] evaluated the main structural and morphological properties of PA12 and their variations during the 3D printing process. By using X-ray diffraction (XRD), differential scanning calorimetry (DSC), and scanning electron microscopy (SEM), the authors explored the powders’ characteristics such as particle size, shape, and surface properties. The results demonstrated that the powder was characterized by low crystallinity and the presence of surface defects. Moreover, an increase in the degree of crystallinity was observed in powder recycled from a previous printing cycle. Stoia et al. [15] evaluated the fracture properties of PA12 through fracture tests on mode I double cantilever beam samples (DCB). The authors indicated that fracture toughness was strongly affected by the presence of internal defects. Further research emphasized that the fabrication process, e.g., powder state, powder particle size, shape, post-fabrication thermal treatment, and process parameters, greatly affected the quality of PA12 parts produced by SLS [18,19,20,21]. Vasquez et al. [18] evaluated the stable sintering region of PA12 fabricated by SLS using thermogravimetric analysis (TGA). In particular, the authors evaluated the degradation energy using TGA analysis and subsequently identified the stable process parameter windows for sintering parts.

This research builds upon previous studies and investigates the fundamental mechanical properties of PA12, which is manufactured using the SLS technique. The objective is to comprehend how the fabrication process and loading conditions affect the mechanical characteristics of 3D-printed components. Understanding the primary factors that impact mechanical properties continues to be a crucial factor for ensuring the widespread adoption of 3D-printed parts in structural applications.

In particular, differential scanning calorimetry (DSC) analyses were carried out on both the powder particles and as-sintered products. A comparison was made between the properties of “as-built” powder (virgin), reused powder (recycled), and their mix. The proposed analysis aims to ascertain the effect of chamber temperature exposure on powder characteristics. Tensile tests were executed to monitor the variations in mechanical properties between different production batches that used varying fractions of recycled powder. Moreover, heat treatments have been shown to be effective in improving the mechanical properties of 3D-printed parts [10,22,23] and reducing residual thermal stresses [23,24,25] that are typical of the 3D printing process. These aspects were also ascertained by subjecting the 3D-printed polyamide to a heat treatment. In addition, the influence of the crosshead displacement rate was evaluated by executing mechanical tests at different displacement rates.

## 2. Materials and Methods

### 2.1. 3D Printing of Polyamide Samples

**Figure 1:**
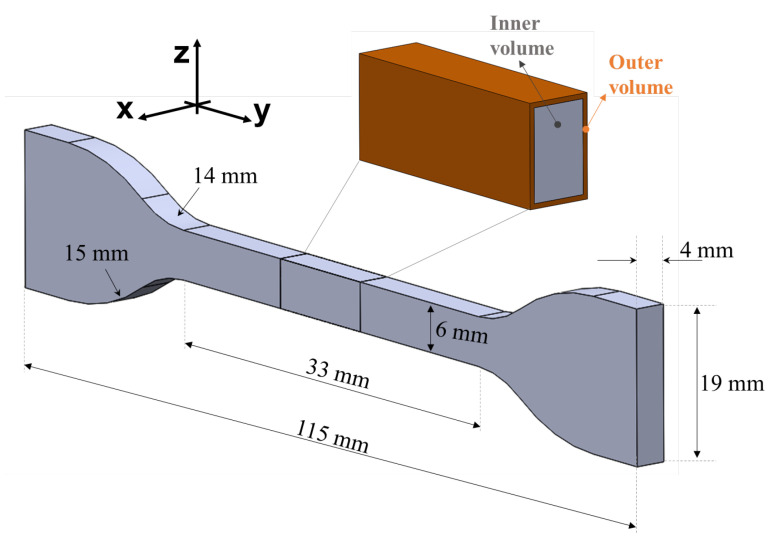
Schematic of dogbone-shaped sample.

The PA12 test samples were fabricated via selective laser sintering (SLS) using the EOS Formiga P110 3D printer (EOS, Krailling, Germany) and PA12 powder (PA2200, EOS, Krailling, Germany) with an average particle size of 56 μm and a 50/50 mixing ratio of virgin and recycled powders. The 3D printing hardware comprised a CO_2_ laser with a spot diameter of 500 μm. The thickness of the working layer employed for 3D printing was equal to 100 μm and the fabrication process occurred in an inert environment (i.e., argon atmosphere in the printing chamber). The main printing parameters are summarized in Table 1. Regarding the laser power and scanning speed, two different values were employed for the inner and outer volumes, as suggested by the 3D printer supplier to improve process efficiency. Specifically, the latter had a 300 μm thick skin, as shown in Figure 1. Dogbone-shaped samples were fabricated according to ASTM-D 638-14 [26] and the dimensions are reported in Figure 1. Overall, three different batches were fabricated and at least five samples were produced in each batch. The samples were fabricated using the same orientation for all production batches. In particular, the building direction, listed as z- in Figure 1, was perpendicular to the loading direction in order to optimize sample distribution on printing volume.

### 2.2. Thermal Analysis

**Figure 2:**
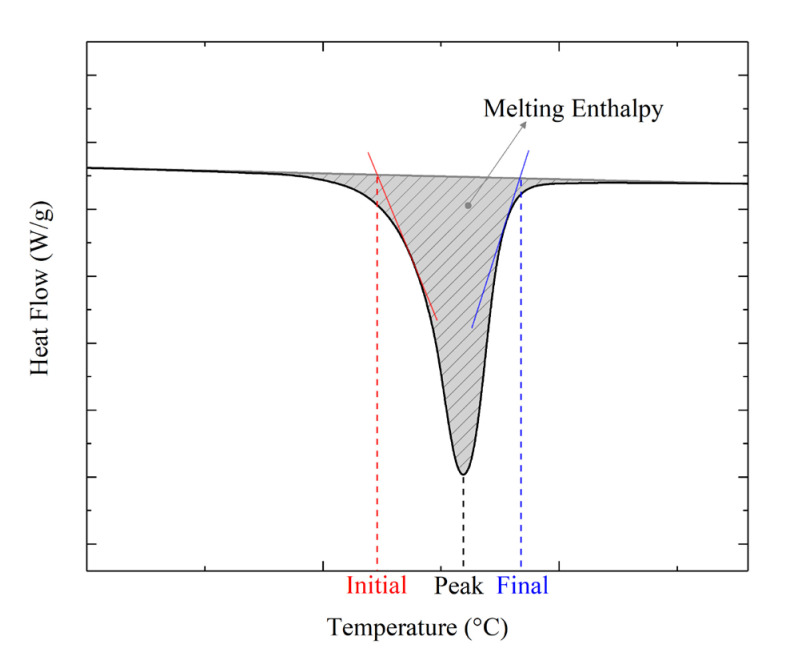
Schematic of characteristic temperatures extracted from heat flow curves and melting enthalpy calculation.

The thermal properties of both powder particles and the sintered parts were evaluated using differential scanning calorimetry (DSC). The samples were heated up to 250 °C from room temperature at a heating rate of 10 °C/min using DSC equipment (DSC 25, TA Instruments, New Castle, DE, USA). From the heat flow curve, one can evaluate both the material enthalpy of fusion and the melting point. The enthalpy of fusion was evaluated as follows: it was equal to the peak area, calculated with a linear baseline between 150 and 220 °C (see Figure 2). The percentage crystallinity, $C\left(\%\right)$, of each sample can be estimated with the following equation:

(1) $$C\left(\%\right)=\frac{∆H_{m}}{∆H_{m,100}}$$

where $∆H_{m}$ is the measured enthalpy of fusion and $∆H_{m,100}$ is the theoretical enthalpy of fusion for 100% crystalline SLS-polyamide-12 (209.3 J/g) [27]. The heat flow curves allowed also us to evaluate the material characteristic temperatures, such as the melting point (i.e., the temperature at which the minimum heat flow value was measured) and the initial and final melting temperatures, which corresponded to melting commencement and completion, respectively. These last temperatures were evaluated as shown in Figure 2 by considering points obtained at the intersection between the horizontal curve and the two tangent lines to the heat flow curve.

Four different types of samples were analyzed: one obtained from a sintered part and three obtained from virgin powder mixed with increasing fractions of recycled powder, i.e., 0% 50%, and 100%. The sintered sample was extracted from a larger part fabricated using a mixture of virgin and recycled powder (50–50) that was not subjected to mechanical loading. At least three samples for each condition were analyzed.

### 2.3. Mechanical Testing

Tensile tests were performed using an electromechanical testing machine, MTS Criterion Model 42 (MTS Systems Corporation, Eden Prairie, MN, USA), equipped with a 5 kN loading cell. Digital image correlation (DIC) was used to measure, in the full field and without contact, the deformation of the specimens during the tensile tests. The system consisted of a GigE camera (Prosilica GT, Allied Vision Technologies GmbH, Stadtroda, Germany) with a 2/3 inches CCD sensor (Sony ICX625, Sony Corporation, Tokyo, Japan), with a pixel size of 3.45 μm, a maximum resolution of 2448 × 2050 pixels, and a maximum frame rate of 15 fps, interfaced with the commercial software Vic-Snap 8 (Correlated Solutions Inc., Irmo, SC, USA). A workstation with the VIC-2D software package (Correlated Solution Inc., Irmo, SC, USA version 2009.2.0) was used to analyze the speckle images. The stress-strain diagrams, Young’s modulus, and Poisson’s ratio were determined using the applied load recorded during the tests and the strain data obtained using DIC.

The tensile tests were carried out at different crosshead displacement rates, selected according to ASTM D638-14 [26], i.e., 0.5, 5, and 50 mm/min. In addition, since the mechanical properties of 3D parts may depend on the presence of residual stress due to the fabrication process, the response of the 3D-printed thermoplastic material after exposure to high temperature was examined. Three-dimensional-printed parts can be affected by different thermal contractions strictly related to different temperature distributions that occur during the process itself. Moreover, post-processing heat treatments have proven to be effective in improving the mechanical properties of 3D-printed parts [10,22,23] and reducing residual thermal stresses [23,24,25]. To this aim, the following annealing treatment was carried out: the bulk specimens were heated up to 100 °C for 1 h in an MTS 651 climatic chamber (MTS Systems Corporation, Eden Prairie, MN, USA). The samples were placed into the preheated climatic chamber and, after the heat treatment, they were slowly cooled down to room temperature (ambient air cooling). The chosen temperature was higher than the glass transition temperature, i.e., around 40 °C [28], but lower than the melting temperature, i.e., 173–180 °C [29]. A temperature of 100 °C was selected according to different studies carried out on other polymeric materials [30,31].

In order to ensure the reliability of the results, at least three specimens were characterized for each displacement rate and thermal treatment.

## 3. Results and Discussion

### 3.1. Thermal Analysis of Powder and Sintered PA

It is well known that the SLS process during the manufacture of parts causes selective melting of powdered materials and that, during the sintering process, the powder surrounding the melted material is subjected to considerable variations in temperature. The tendency to reuse the recovered non-melted powders for the production of new parts is widespread, especially for reducing waste and process costs [32,33]. Therefore, it is very important to understand the influence of thermal exposure on the properties of powders.

**Figure 3:**
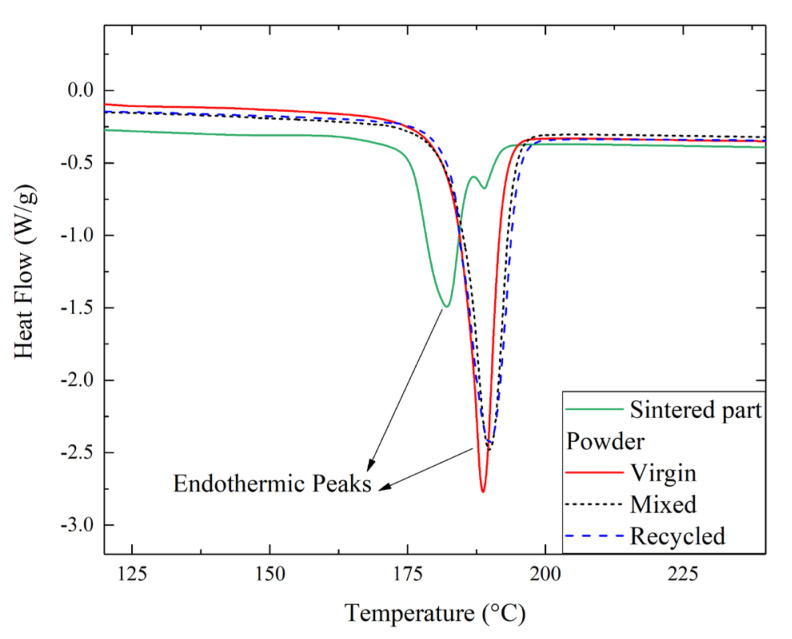
Heat flow curves for the sintered part and powder samples (virgin, recycled, and mixed).

To study the effect of temperature exposure on the powder, differential scanning calorimetry (DSC) analysis was performed by the authors on both the sintered parts (produced with 50–50% mixed powders) and the powders. Regarding the 3D-printed parts, the recycling of powder is recommended by the producer to reduce cost and waste. However, the amount of recycled powder should be limited to avoid compromising quality. For this reason, it was chosen to use an equal share, i.e., 50–50%, of recycled and virgin powders. Regarding the powder, different conditions have been studied with different percentages of recycled powder, i.e., 0%, 50%, and 100%. These samples are named virgin, mixed, and recycled, respectively. The heat flow curves are shown in Figure 3.

The result revealed a significant variation in melting temperature after sintering. In fact, the sintered part (50/50) was characterized by a lower peak value, i.e., 182 °C. A similar value was also measured by Messiha et al. [28] and Dadbakhsh et al. [34] for laser-sintered parts realized by PA2200. Moreover, as observed in Figure 3, Dadbakhsh et al. [34] also observed the occurrence of two different peaks for the sintered part, which was attributed to the inhomogeneous melting of the sintered material.

**Figure 4:**
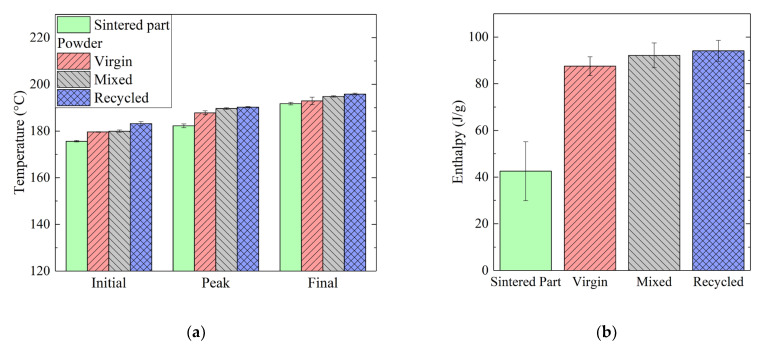
(**a**) Initial, peak, and final temperatures extracted from heat flow curves as shown in Figure 2. (**b**) Melting enthalpy of a sintered part as compared to virgin, recycled, and mixed powders.

On the other hand, the curves relating to the powder exhibited comparable behaviors, regardless of composition, with melting temperatures between 187 °C (for virgin powder) and 190 °C (for recycled powder). Similarly, Cai et al. [35] obtained a value of 188.9 °C for the melting temperature of PA2200 powder. In analyzing the characteristic temperatures, however, some differences were detected, as shown in the bar graph of Figure 4a. In particular, in addition to peak temperature, the initial and final melting temperatures were evaluated.

It can be noted that the temperatures detected on the sintered sample were always lower than the temperatures detected on the powders and that, for the latter, the three temperatures increased as the percentage of recycled powder increased (0%, 50%, 100%). Moreover, regarding the sample consisting of mixed powder, it was found that the initial melting temperature was similar to that measured for the virgin sample, while the final melting temperature was similar to that measured for the recycled sample. Consequently, the melting range was greater for the mixed powder.

The presence of recycled powder in the powder bed can increase the melting temperatures of the mixture with the consequent risk of favoring the creation of lack-of-fusion defects during sintering. Thus, it could be necessary to adjust the printing parameters, such as laser power or chamber temperature as a function of the percentage of recycled powder employed for part production (sintered part).

The melting enthalpy values obtained for all samples are reported in the graph of Figure 4b. A significant difference between the sintered part and the powder samples was also found. In particular, this latter showed higher enthalpy values, i.e., between 88 J/g (for virgin powder) and 94 J/g (for recycled powder), compared to the results of the printed part, i.e., 43 J/g. Similar values were obtained for powder samples by Dadbakhsh et al. [34] and Cai et al. [35], thus demonstrating the reliability of powder feedstock production. Moreover, Cai et al. [35] also observed a reduction in both melting temperature and enthalpy after the printing process.

Using Equation (1) reported in the previous section, the degree of crystallinity was evaluated and a value of 20% was found for the sintered part, while degrees of crystallinity of 42%, 44%, and 45% were calculated for the virgin, mixed and recycled powders. A similar increase in powder crystallinity degree after aging was observed by Dadbakhsh et al. [34]. This variation was attributed to an increase in crystal growth, promoted by the exposure of powder particles to high temperatures during sintering. However, after the sintering process, the crystalline chains were modified, thus leading to a significant reduction in the measured degree of crystallinity observed in PA12 sintered parts.

### 3.2. Results of Quasi-Static Tensile Tests

The mechanical properties of PA12 were evaluated. The experimental tests were performed according to ASTM-D 638-14 [26]. A 0.5 mm/min displacement rate was selected to minimize potential rate-dependent effects due to the viscoelastic nature of the nylon substrates.

**Figure 5:**
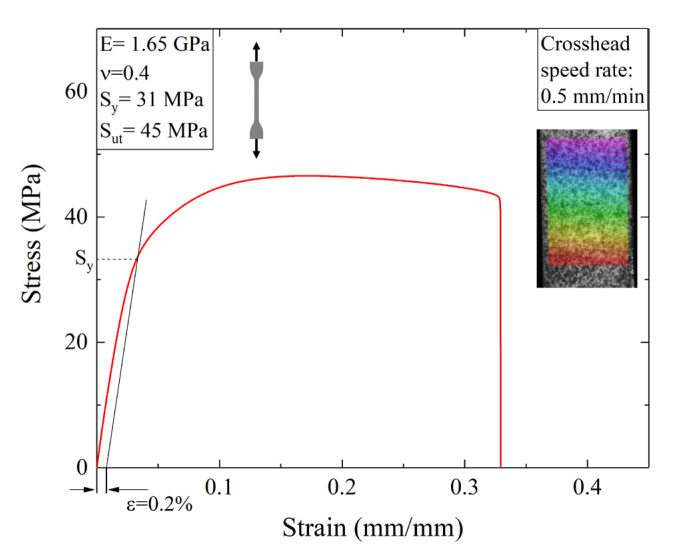
A typical stress–strain curve obtained during tensile testing on nylon specimens. The inset represents the y-displacement field.

A typical example of the engineering stress-strain curve is shown in Figure 5. In the same graph, the displacement field in the y-direction (see Figure 1) obtained using DIC is also reported. The results clearly indicated that the 3D-printed material exhibited an initial linear elastic response followed by strain hardening and extensive plasticity before the final fracture. The yield strength (i.e., S_y_), ultimate tensile strength (i.e., S_ut_), and strain at break were evaluated from the stress-strain plots while Young’s modulus and Poisson’s ratio were obtained using DIC strain maps.

It should be noted that the tensile strength was slightly lower than that reported by the manufacturer [29]. This difference could be attributed to a mismatch in the strain rate used for the experimental tests, although the rate used by the manufacturer was not disclosed. Moreover, the mechanical properties are also dependent on the internal structure of the sample, e.g., porosity or inner defects [36,37]. It is well known that parts obtained by additive manufacturing are characterized by the occurrence of internal porosity [38,39,40,41,42]. In a previous work by the authors [40], the inner morphology of 3D-printed dogbones fabricated using PA12 was examined, revealing a significant level of porosity at approximately 3%.

Specimens from different printed batches were tested to identify any batch-to-batch variations in yield strength, ultimate strength, and strain at break. Specifically, we analyzed three separate batches that were produced at different times, ensuring a minimum interval of three months between each batch. Within each batch, we fabricated and tested a minimum of three samples.

**Figure 6:**
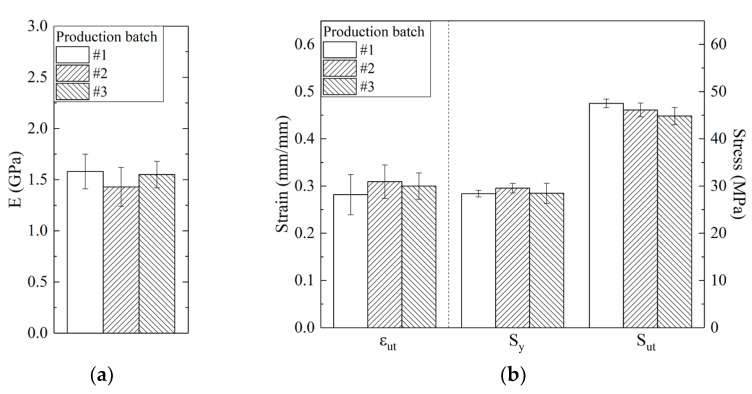
Assessment of batch-to-batch variations for (**a**) Young’s Modulus and (**b**) elongation at break, yield strength, and ultimate tensile strength.

The results are shown in Figure 6 and do not display significant changes, although the resulting stress-strain curves showed some degree of variation within a given batch, especially concerning the elongation at break.

**Figure 7:**
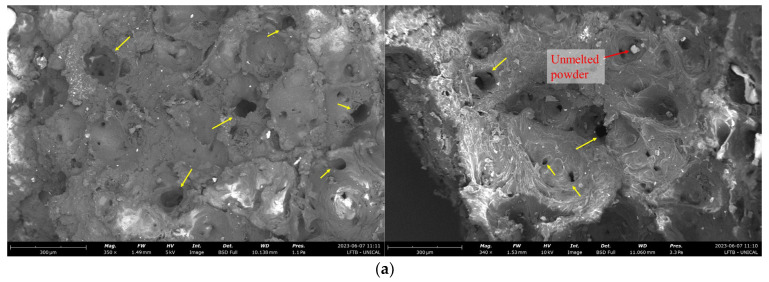

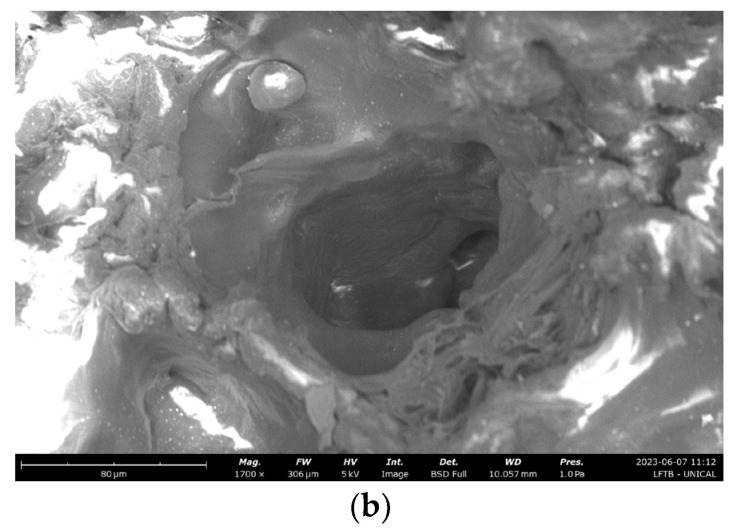
(**a**) Micrographs of fracture surfaces and (**b**) enlarged image of pore detected on the fracture surface. Yellow arrows show the main pores detected on fracture surfaces.

It is important to highlight that sudden final failure, i.e., brittle failure, occurs in all mechanical tests. This result suggests that sample morphology is quite different from that of polymeric parts fabricated by traditional techniques, e.g., injection molding. Indeed, while the deformation process highlighted a ductile behavior, we did not observe any strain localization and necking before fracture. However, the fracture surfaces revealed the occurrence of dimples and porosity defects, similar to those observed by Yao et al. [43]. Figure 7 displays SEM images of the fracture surfaces, revealing the existence of significant porosity. Notably, certain pores stood out due to their considerable dimensions, as depicted in Figure 7b. Moreover, observable unmelted powder particles were believed to have a substantial influence on the porosity formation process. Typically, pores originate during the SLS printing process, as testified by different works [40,44,45]. Moreover, some big dimples originate after fracture due to the detachment of unmelted particles [43].

### 3.3. Effect of Crosshead Displacement Rate on the Mechanical Properties of 3D-Printed Nylon

**Figure 8:**
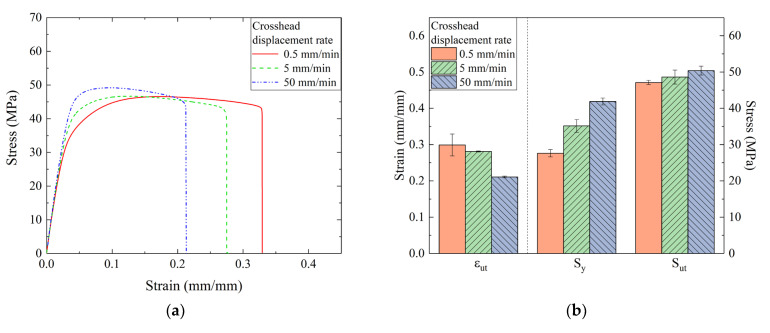
(**a**) Stress-strain curves for dogbone-shaped samples tested at different crosshead displacement rates; (**b**) comparison between the yield strength, ultimate strength, and ε at break obtained under different displacement rate conditions.

As already mentioned, due to potential viscoelastic effects, the mechanical properties of the polymer are strictly dependent on the crosshead displacement rate and, for this reason, additional tensile tests were conducted at 0.5, 5, and 50 mm/min. Representative stress-strain plots corresponding to these crosshead displacement rates are shown in Figure 8a, while a bar chart of the main mechanical parameters is reported in Figure 8b.

The results demonstrated that laser-sintered nylon displayed the expected rate dependency, similar to traditionally processed polymers. As the crosshead displacement increased, the elongation at break decreased while the yield strength progressively increased. However, the differences became more notable by changing the displacement rate from 5 to 50 mm/min. For displacement rates <5 mm/min. it appeared that viscoelastic effects did not heavily affect material behavior. As for ultimate strength, our results indicated a similar outcome for any of the crosshead displacement rates. The obtained mechanical properties were compared with studies of PA specimens fabricated by SLS under similar processing conditions (e.g., energy density, build orientation) [19,46,47] and with results obtained for samples fabricated using other techniques, i.e., injection molding [48] and multi-jet fusion (MJF) [35,46] and main data are reported in Table 2. The results obtained for the 3D-printed samples were comparable [19,35,46,48], even if Razaviye et al. [46] found a quite lower value for ultimate strain. With regard to the results obtained using different fabrication processes, Meyer et al. [47] found higher ultimate strength for injection-molded PA12. The authors found that by properly selecting the melt temperature, the maximum stress could be increased to values higher than 60 MPa. Moreover, Razaviye et al. [46] found that one could improve the tensile strength by tailoring the printing parameters. The authors achieved values above 60 MPa once the process was properly tuned, thereby suggesting that the removal of internal defects such as porosity is a key point for achieving mechanical properties similar to standard injection-molded PA12.

### 3.4. Effect of Post-Processing Thermal Treatment on the Mechanical Properties of 3D-Printed Nylon

**Figure 9:**
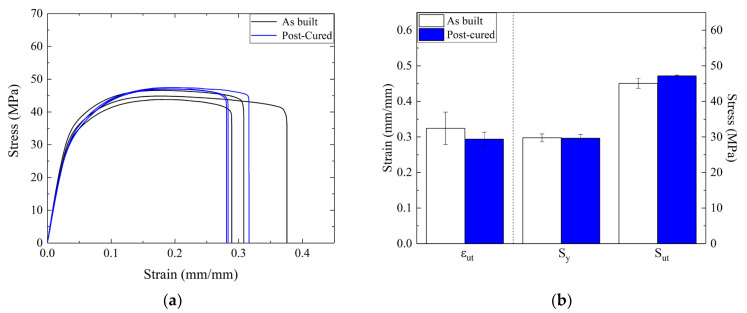
Influence of thermal treatment on (**a**) the stress-stain curve and (**b**) the yield strength, ultimate strength, and ε at break.

High temperature exposure can modify the mechanical properties of parts made by SLS, thereby reducing residual stresses due to heat shrinkage [31,49,50]. Different studies focused on materials obtained using fused deposition modeling (FDM) demonstrated that heat treatment can improve tensile strength thanks to the possibility of enhancing the bonding strength between raster layers and promoting recrystallization [31,51]. However, it was crucial to evaluate whether the properties of the 3D-printed material could be affected by high temperature curing, considering that the mechanisms involved may differ for SLS and FDM samples. Three dogbone-shaped samples were subjected to isothermal heating at 100 °C for 1 h in a climatic chamber (MTS 651 MTS Systems Corporation, USA) and subsequently gradually cooled down to room temperature. After that, tensile tests were conducted and the results are reported in Figure 9. No dimensional or geometrical variances were observed following the thermal treatment. However, it should be noted that the dogbone-shaped samples lacked intricate features and, as a result, the impact of the thermal treatment on the shapes of these details needs to be evaluated using specially fabricated samples.

It was observed that heat treatment seemed to reduce the experimental variability, which was quite noteworthy for the elongation at break and, to a smaller extent, the yield and ultimate stresses. The dogbones that were not subjected to heating were characterized by some differences in the post-yielding of the stress-strain curve, even if the elastic behavior and the yield strength seemed to be not affected by the thermal treatment, i.e., the yield stress value was unvaried. Overall, differences in the stress-strain curves before and after the thermal treatment were observed only in the plastic region. The experimental results demonstrated a reduction in the measured ultimate strain, around 9.5%, and a small increment in the ultimate strength, approximately equal to 4.6%. Similar results were obtained by Akhoundi et al. [31] for HTPLA samples subjected to heat treatment at 110 °C for 1 h. In particular, the authors observed an increase in tensile strength between 7% and 9% for samples subjected to heat treatment. This increment was attributed to material recrystallization. It was found that a change in sample appearance, i.e., from transparent to nearly white, was related to the increase in crystallinity percentage. Moreover, other authors observed an increase in ultimate strength and crystallinity after annealing treatments on different polymers [23,25]. Overall, the increase in the crystallinity percentage led to a modification of the sample’s mechanical behavior in that it appeared to be more brittle, thus resulting in a lower ultimate strain value, as observed in this work.

## 4. Conclusions

This study involved an evaluation of the key mechanical properties and associated challenges pertaining to PA12 3D-printed components. The thermal characteristics of the material were evaluated using the DSC technique, encompassing the analysis of sintered parts and powder particles. The results revealed a significant difference between the thermal properties of sintered parts and the powder particles, with sintered samples exhibiting a reduction of approximately 5 °C in melting temperature. Furthermore, a comparative analysis was conducted to examine the properties of virgin, recycled, and mixed powders, indicating a slight increase in melting temperature as the percentage of recycled powder in the sample increased. This variation could heighten the risk of fusion defects due to inadequate selection of process temperature and laser power. Further investigation is needed to clarify this aspect.

The primary mechanical properties of the 3D-printed specimens were determined through tensile tests carried out on separate production batches. The results exhibited minimal variability, thus demonstrating the reliability of the 3D printing process. The influence of displacement rate was additionally assessed by conducting tests at different rates, revealing a noticeable viscoelastic behavior. Specifically, increasing the displacement rate led to higher yield stress and lower deformation at break values. Lastly, the impact of thermal treatment on the mechanical properties of PA12 was evaluated. The findings demonstrated a slight variation in the main mechanical properties, with a reduction of approximately 9.5% in ultimate strain and a marginal increase of around 4.6% in ultimate strength, which could be attributed to material recrystallization

## Figures and Tables

**Table 1:** Printing parameters.

Printing Parameters	Inner Volume	Outer Volume
Laser power [W]	21	16
Scanning speed [mm/s]	2500	1500
Hatching scan spacing [mm]	0.25	
Energy density [mJ/m^2^]	33.6	42.7
Layer thickness [mm]	0.1	
Chamber temperature [°C]	168	
Powder composition	50% virgin–50%recycled	

**Table 2:** Mechanical properties obtained for PA12 samples at different displacement rates in comparison with results obtained in other works [19,35,46,47,48].

	Production Technology	Displacement Rate [mm/min]	E [GPa]	S_y_ [MPa]	S_ut_ [MPa]	Ultimate Strain
This work	SLS	0.5	1.65	27.6	47.1	29.9
		5	1.68	35.1	48.6	28.1
		50	1.66	41.9	50.4	21.1
Meyer et al. [47]	Injection molding	50	1.01 ÷ 1.09	35.6 ÷ 36.9	53 ÷ 68	180 ÷ 330
Pavan et al. [19]	SLS	2	1.68	-	47.1	14.7
Abbott et al. [48]	SLS	2	1.71	-	45.8	18.2
	MJF		1.51	-	44.2	1.51
Razaviye [46]	SLS	5	1.31	-	43.39	5.65
Cai et al. [35]	SLS	10	1.61	-	43.9	26.6
	MJF		1.39	-	44.5	15.9

## Data Availability

The data reported in this work are available upon request.
